# Sex and Age Effects of Functional Connectivity in Early Adulthood

**DOI:** 10.1089/brain.2016.0429

**Published:** 2016-11-01

**Authors:** Chao Zhang, Nathan D. Cahill, Mohammad R. Arbabshirani, Tonya White, Stefi A. Baum, Andrew M. Michael

**Affiliations:** ^1^ Autism and Developmental Medicine Institute, Geisinger Health System, Lewisburg, Pennsylvania.; ^2^ Chester F. Carlson Center for Imaging Science, Rochester Institute of Technology, Rochester, New York.; ^3^ School of Mathematical Sciences, Rochester Institute of Technology, Rochester, New York.; ^4^ Institute for Advanced Application, Geisinger Health System, Danville, Pennsylvania.; ^5^ Department of Child and Adolescent Psychiatry, Erasmus MC-Sophia Children's Hospital, Rotterdam, The Netherlands.; ^6^ Faculty of Science, University of Manitoba, Winnipeg, Manitoba, Canada.

**Keywords:** age effects, early adulthood, fMRI, functional connectivity, resting-state fMRI, sex effects

## Abstract

Functional connectivity (FC) in resting-state functional magnetic resonance imaging (rs-fMRI) is widely used to find coactivating regions in the human brain. Despite its widespread use, the effects of sex and age on resting FC are not well characterized, especially during early adulthood. Here we apply regression and graph theoretical analyses to explore the effects of sex and age on FC between the 116 AAL atlas parcellations (a total of 6670 FC measures). rs-fMRI data of 494 healthy subjects (203 males and 291 females; age range: 22–36 years) from the Human Connectome Project were analyzed. We report the following findings. (1) Males exhibited greater FC than females in 1352 FC measures (1025 survived Bonferroni correction; $p  <  7.49{ \rm{E}} - 6$). In 641 FC measures, females exhibited greater FC than males but none survived Bonferroni correction. Significant FC differences were mainly present in frontal, parietal, and temporal lobes. Although the average FC values for males and females were significantly different, FC values of males and females exhibited large overlap. (2) Age effects were present only in 29 FC measures and all significant age effects showed higher FC in younger subjects. Age and sex differences of FC remained significant after controlling for cognitive measures. (3) Although *sex* $\times$ *age* interaction did not survive multiple comparison correction, FC in females exhibited a faster cross-sectional decline with age. (4) Male brains were more locally clustered in all lobes but the cerebellum; female brains had a higher clustering coefficient at the whole-brain level. Our results indicate that although both male and female brains show small-world network characteristics, male brains were more segregated and female brains were more integrated. Findings of this study further our understanding of FC in early adulthood and provide evidence to support that age and sex should be controlled for in FC studies of young adults.

## Introduction

Resting-state functional magnetic resonance imaging (rs-fMRI) is a powerful neuroimaging technique that enables researchers to measure spontaneous fluctuations in activity between distinct brain regions (Biswal et al., 1995). Unlike task-based fMRI, rs-fMRI does not require participants to be trained in specific tasks, and therefore, results are not confounded by task performance. Functional connectivity (FC) measured through rs-fMRI is utilized to explore the brain's intrinsic functional networks (Biswal et al., 2010), and the presence of consistent functional networks has been replicated across numerous studies (Allen et al., 2011; Damoiseaux et al., 2006, 2008; Shehzad et al., 2009; Smith et al., 2009; Zuo et al., 2010).

Intrinsic FC can be used as a tool for human connectomics (Van Dijk et al., 2010), and variability of resting-state networks may be useful for characterizing both normal and abnormal brain function. Differences in resting-state FC compared to healthy controls have been found in autism (Muller et al., 2011), attention-deficit/hyperactivity disorder (Uddin et al., 2008), Alzheimer's disease (Li et al., 2013), unipolar depression (Anand et al., 2009), epilepsy (Wurina et al., 2012), and schizophrenia (Jafri et al., 2008; Venkataraman et al., 2012). Although consistent FC group differences have been found between patients with neurodevelopmental/neuropsychiatric disorders and matched controls, classification of patients based on FC measures has proven to be a difficult task (Arbabshirani et al., 2013). This difficulty emphasizes the need for better characterization of FC in healthy populations before extending FC research to atypical populations. In this study, we characterize the effects of sex and age on FC in healthy young adults.

Sex plays an important role in FC, but conclusions regarding sex effects are not well established. Males and females have been shown to differ in various connectivity analyses. Bluhm et al. (2008) examined the default mode network (DMN) and detected stronger FC for females within the posterior cingulate cortex/precuneus and the bilateral medial prefrontal cortex, whereas no brain region exhibited greater FC for males. Another study using independent component analysis (ICA) by Allen et al. (2011) performed a statistical comparison between sexes on frequency composition, spatial map, and functional network connectivity measures. Although sex effects were not found to be as extensive as aging effects, specific ICA components (in auditory, sensorimotor, and attentional networks) did show significant sex differences. Tian et al. (2011) applied graph theoretical analysis on 90 Automatic Anatomical Labeling (Tzourio-Mazoyer et al., 2002) atlas regions and reported that compared to females, males had higher clustering coefficients in the right hemispheric networks but lower clustering coefficients in the left hemispheric networks. Sex-related differences in the developmental trajectories of functional homotopy (Zuo et al., 2010) and lateralization (Liu et al., 2009) have also been examined. Despite these findings, sex effects on rs-fMRI FC remain inconclusive and in some cases contradictory; while Biswal et al. (2010) found consistent sex variations of FC using three distinct methods (seed-based, fractional amplitude of low-frequency fluctuations, and ICA), Weissman-Fogel et al. (2010) found no significant differences between sexes in FC and therefore reported no need to control for sex for rs-fMRI studies. Therefore, further effort is required to derive a clear understanding of sex effects.

Similarly, the effects of age on FC are not well characterized. Previous studies have examined the heterogeneous effects of age-related differences in FC at different developmental stages from the fetus *in utero* (Thomason et al., 2015) to elderly populations (Bernard et al., 2013; Madden et al., 2010; Seidler et al., 2015). During fetal development, primitive forms of motor, visual, default mode, thalamic, and temporal FC networks were observed. Increased long-range cerebral–cerebellar, cortical–subcortical, and intrahemispheric FC were discovered during gestation at 24–38 weeks (Thomason et al., 2015). Disrupted FC in elderly populations has been reported in the corticocerebellar network (Bernard et al., 2013), in the DMN (Xiao et al., 2015), and in the motor system network (Langan et al., 2010; Seidler et al., 2015). Furthermore, multiple studies have reported that FC in the DMN may be most susceptible to aging effects (Bluhm et al., 2008; Campbell et al., 2013; Damoiseaux et al., 2008; Esposito et al., 2008). Aging effects on FC have been studied using various methods such as ICA, seed-based analyses, region of interest (ROI)-based analyses, and graph analyses (Dennis and Thompson, 2014). However, findings regarding age differences are not well established and many studies are based on small cohorts with less than 100 subjects. In addition, age-related FC variability studies most often compare two distinct age groups (Bernard et al., 2013; Wu et al., 2011) between adolescents and middle-aged adults or elderly populations (Andrews-Hanna et al., 2007; Evers et al., 2012; He et al., 2013; Shaw et al., 2015). Therefore, it is not clear if previously reported age effects of FC emerge in early adulthood. To our knowledge, no previous studies have examined full brain FC in early adulthood using high-quality images on a large number of subjects and this study will attempt to address this knowledge gap.

The primary goal of this article is to examine rs-fMRI FC of the whole brain between ROIs as defined by the AAL atlas using data from the human connectome project (HCP). We aim to test and identify sex and age effects on FC by linear regression. In addition, local and global brain graph properties will be derived to explore differences in brain organization between males and females.

## Materials and Methods

### Data acquisition and preprocessing

This study includes 494 healthy adults (203 males and 291 females, age: 22–36 years) from the first rs-fMRI run (Session 1, phase encoding in a left-to-right direction) of HCP S500 release (db.humanconnectome.org). Subject demographics and behavioral measures are presented in Table 1, including two-sample *t*-test *p*-values between sexes for four demographics and seven cognitive scores. The seven cognitive scores were selected based on the NIH cognition battery toolbox (www.nihtoolbox.org).

**Table 1.** Subject Demographics and Cognitive Measures (*N* = 494)

	*Male (*N = *203) mean (SD)*	*Female (*N = *291) mean (SD)*	p
Age (years)	29.0 (3.5)	29.4 (3.4)	0.24
Education (years)	14.7 (1.9)	14.9 (1.9)	0.18
Income^a^	5.1 (2.2)	5.0 (2.2)	0.68
Right-handedness ratio^b^	185/203 = 0.91	261/291 = 0.90	0.60
Executive function/inhibition	104 (10)	102 (9)	0.11
Executive function/cognitive flexibility	103 (10)	103 (10)	0.91
Episodic memory	101 (17)	107 (17)	9E-5^c^
Working memory	103 (14)	102 (14)	0.23
Processing speed	99 (17)	101 (16)	0.15
Language/vocabulary comprehension	109 (15)	106 (15)	0.02^c^
Language/reading decoding	108 (15)	104 (15)	0.01^c^

Cognition scores are age adjusted based on the NIH cognition battery toolbox.

^a^ Total household income categories: <$10,000 = 1, 10K–19,999 = 2, 20K–29,999 = 3, 30K–39,999 = 4, 40K–49,999 = 5, 50K–74,999 = 6, 75K–99,999 = 7, ≥100,000 = 8.

^b^ Schachter et al. (1987).

^c^ Indicates statistical significance for *p* < 0.05.

SD, standard deviation.

All HCP rs-fMRI data were acquired on a Siemens Skyra 3T scanner housed at Washington University in St. Louis. MR imaging protocols are described in the S500 release manual available at db.humanconnectome.org. During the resting scans, participants were asked to keep their eyes open with relaxed fixation on a projected bright cross-hair on a dark background. Duration of the scan was 14 min 33 sec with a TR of 0.72 sec, equating to 1200 volumes.

The preprocessed data we downloaded had undergone standard preprocessing steps (such as motion correction and spatial normalization) and ICA denoising to remove non-neural spatiotemporal components (Griffanti et al., 2014; Salimi-Khorshidi et al., 2014). In addition, the 24 head motion parameter (Satterthwaite et al., 2013) time series were high-pass filtered and were then regressed out of the data. To confirm that head motion did not contribute to FC sex differences, we calculated two-sample *t*-values of the frame displacement (Power et al., 2012, 2014) between males and females and found no significant differences. Similarly, we calculated the correlation between frame displacement and age and found that there was no significant association.

### Linear regression and graph theoretical analyses

**FIG. 1.**
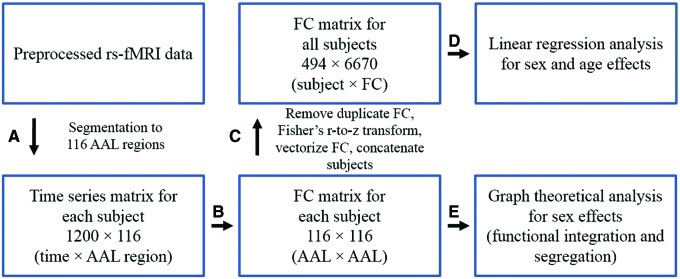
Steps of FC analyses. **(A)** Preprocessed rs-fMRI data were parcellated using the AAL atlas into 116 brain regions. **(B)** Pearson correlation was calculated for each subject's time series to obtain a 116 × 116 FC matrix. **(C)** FC of all subjects were concatenated to derive a group FC matrix. **(D)** Linear regression analysis was applied to the group FC matrix to identify sex and age effects on FC. **(E)** Group comparisons between sexes were implemented for graph properties of two categories: functional integration and segregation. FC, functional connectivity; rs-fMRI, resting-state functional magnetic resonance imaging. Color images available online at www.liebertpub.com/brain

The workflow of this study is shown in Figure 1.

Step A: Preprocessed rs-fMRI data were downloaded from the HCP site (db.humanconnectome.org). To explore the brain networks at the macrolevel, the AAL atlas was utilized to segment each subject's whole-brain rs-fMRI into 116 regions (90 cortical/subcortical regions [45 for each hemisphere] and 26 cerebellar/vermis regions). The AAL segmentation methodology has previously been applied in various brain imaging studies (Park et al., 2013; Shirer et al., 2012; Xu et al., 2015). The list of brain regions contained in the AAL atlas is provided in Supplementary Table S1 (Supplementary Data are available online at www.liebertpub.com/brain). Within each AAL region, an average time series was calculated.

Step B: From the time series matrix of size 1200 × 116 (time points × AAL regions), FC matrices (116 × 116) were derived for each subject by applying Pearson correlation across the whole duration of the time series. For better interpretation, the 116 regions were reordered and grouped into 7 brain lobes according to the hierarchical clustering of AAL brain regions implemented by Salvador et al. (2005).

Step C: The number of FC was reduced from 116 × 116 = 13,456 to 6670 by removing duplicate FC present in the symmetric FC matrix. All FC were then Fisher's *z*-transformed, rearranged to FC row vectors, and the FC row vectors were stacked across subjects. A 494 × 6670 (subjects × FC) group FC matrix across all subjects was constructed for subsequent regression analysis.

Step D: Regression analysis was applied to each column of the group FC matrix. The initial full model included *sex*, *age*, and *sex* × *age* interaction as covariates:

\begin{align*}
{F_i} = \beta _i^0 + \beta _i^s \ sex + \beta _i^a \ age + \beta _i^{{int} } \ sex \times age + { \varepsilon _i} \tag{1}
\end{align*}

where *F_i_* is the FC vector for a pair of AAL regions across subjects. $i = 1 , 2 , \ldots 6 , 670$ corresponds to all possible combinations of AAL regions. ${ \beta _i}$'s are the regression coefficients and ${ \varepsilon _i}$ is the error term.

The backward stepwise approach (Ronald Christensen, 2001) was applied to select the best model for each FC. We used this scheme since using one model (same set of covariates) for all 6670 FC is not appropriate due to the fact that not every FC measure may incorporate the effects of all the potential covariates. In each round, the backward stepwise approach calculates a significance *p*-value quantifying the effect of removing each covariate of the current model. It then removes the covariate that had the most insignificant effect in each iteration until any further reduction would exert significant difference in the *F*-statistic of the model compared to the model at a previous iteration. This approach yields five possible models and they are as follows: M1: no covariates, M2: *sex* only, M3: *age* only, M4: *sex* and *age*; M5: *sex*, *age*, and *sex* × *age*. The *p*-values of regression coefficients in each model were used to identify the significant effects on FC. To explore how the behavioral measures would affect the sex and age effects on FC, in a separate analysis, seven cognitive measures listed in Table 1 were added to the regression models determined by the backward stepwise model selection.

Step E: Each 116 × 116 FC matrix was thresholded and converted to a binary adjacency matrix for graph theoretic analyses. Graph measures are dependent on the total cost of the network, for example, the network clustering coefficient and global efficiency increase monotonically as edges are added to a graph. Therefore, to ensure the most direct mathematical comparability of graph properties across subjects, a proportion threshold (Bassett et al., 2012; Bullmore and Bassett, 2011) based on graph density was applied to each FC matrix, where density threshold ranged from 0.05 to 0.95 at 0.05 intervals. For example, when a density threshold of 0.1 is applied, for each subject, the top 10% of the FC are retained and the FC matrix is converted to a binary adjacency matrix. Different thresholds were applied to compare group differences of graph properties at various graph densities. This thresholding scheme was reported to be more stable compared to absolute (correlation-based) thresholds (Garrison et al., 2015).

Network measures in this study were derived using the Brain Connectivity Toolbox (Rubinov and Sporns, 2010). Nodal clustering coefficient and nodal local efficiency were adopted to examine the regional characteristics of the functional brain network. The clustering coefficient of a node is a measure of the degree to which that node in the graph tends to cluster together with its neighboring nodes. The local efficiency quantifies how well information is exchanged within that neighborhood. For the global network matrices, the network clustering coefficient (*C*) and the characteristic path length (*L*) were calculated. These are two key graph parameters that can also characterize the small-world organization of a network. *C* is the average of the nodal clustering coefficients across the nodes. *L* is the average of shortest path length between all pairs of nodes and quantifies the number of processing steps for information transfer across the brain. To handle the possible infinite path lengths between disconnected nodes, *L* was calculated as the harmonic mean of geodesic distances (Latora and Marchiori, 2001). The number of nodes included in the largest connected component was calculated for different proportion thresholds. The small-worldness metric of a network ($\sigma$) was then estimated as the ratio between the normalized clustering coefficient and the normalized characteristic path length: $\sigma = { \frac { C / { C_ { rand } } }  { L / { L_ { rand } } } }$ (Humphries and Gurney 2008), where ${C_{rand}}$ and ${L_{rand}}$ are the average of *C* and *L* derived from 30 corresponding random networks generated by rewiring each edge approximately 10 times while preserving the original degree distribution (Maslov and Sneppen, 2002; Rutter et al., 2013; Wang et al., 2010). Compared to a random network, a small-world network has a similar *L* value but a higher *C* value. Therefore, if the value of $\sigma$ is greater than 1, a network is considered to exhibit small-world characteristics. Graph properties for both individual nodes and the whole-brain network were compared between males and females by two-sample *t*-tests.

## Results

### Linear regression model

**FIG. 2.**
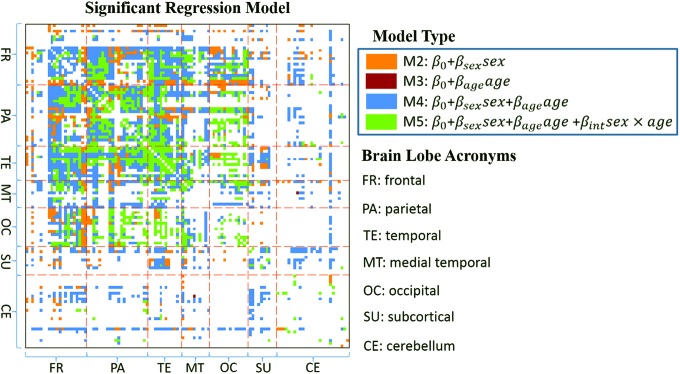
Significant regression models with *F*-statistic $p  < 7.49{ \rm{E}} - 6$) for all ROI pairs are color coded according to the best model fit. The above *p-*value corresponds to Bonferroni corrected threshold at $\alpha = 0.05.$ The 116 AAL regions are grouped into 7 brain lobes separated by red dash lines. ROI, region of interest. Color images available online at www.liebertpub.com/brain

FC measures with significant regression models (*F*-statistic $p < 7.49{ \rm{E}} - 6$ corresponding to Bonferroni threshold at α = 0.05) are presented in Figure 2, where the corresponding model of an FC is color coded. Regression models for 1994 of 6670 FC measures ($30\%$) were significant. Out of the 1994 significant models, M2–M5 are chosen 321 ($16\%$), 1 ($<  0.1\%$), 1026 ($51\%$), and 646 (32$\%$) times, respectively. This indicates that FC variability is best captured by M4 (*sex* and *age*) and M5 (*sex*, *age*, and *sex* × *age*) for the majority of the FC measures. M2, for which the model only contains the *sex* covariate is the next best fit, while M3 for which the model contains *age* only is selected just once. In Figure 2, we also note that out of the 1994 significant FC models, 938 ($47\%$) are present in the top-left $3 \times 3$ block, which contains the frontal, parietal, and temporal lobes.

### Sex and age effects of functional connectivity

**FIG. 3.**
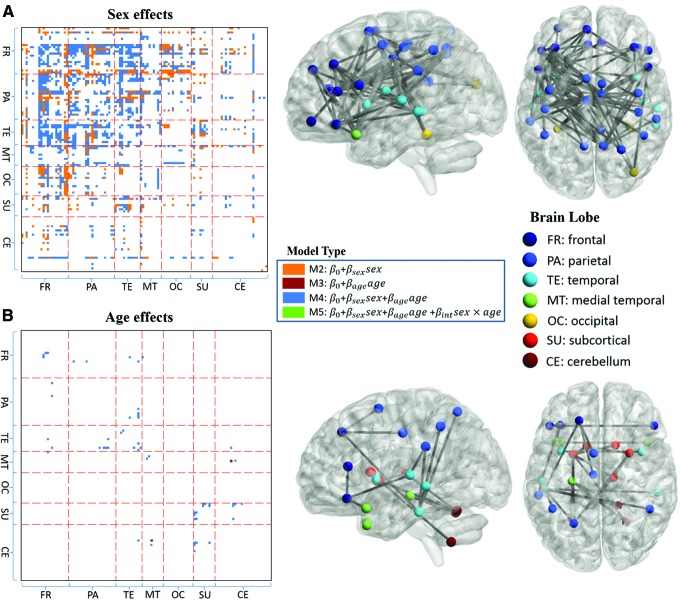
ROI pairs with significant **(A)** sex effects and **(B)** age effects ($p < 7.49{ \rm{E}} - 6$ corresponding to Bonferroni threshold at α = 0.05) on FC are presented, in the FC matrix (left) and on brain map (right). In the matrix plots, ROI pairs with significant sex and age effects are color coded to indicate the model from which the significance of the covariates is derived. For the brain map, 100 ROI pairs with the most significant sex effects are presented for visualization and for the age effects, all surviving pairs are presented. Color images available online at www.liebertpub.com/brain

For FC measures with significant regression models, the significance of *sex*, *age*, and *sex* $\times$ *age* covariates is further explored. Under Bonferroni correction at $\alpha = 0.05$, there is no significant *sex* $\times$ *age* interaction. ROI pairs with significant sex or age effects are shown in Figure 3. Out of the 1994 FC measures that have significant model fits based on the *F*-statistic, in 1352 FC measures, males have higher FC than females and 641 females have higher FC than males. Out of the 1352 FC measures where males show higher FC, 1025 are significant after Bonferroni correction ($p  <  7.49{ \rm{E}} - 6$), but out of the 641 measures where females show higher FC than males, none are significant after Bonferroni correction (Fig. 3A). FC measures that are higher for females failed to survive even at a more lenient threshold of *p* < 0.001 uncorrected. Age effects are less widespread; only 29 FC measures have a significant relationship with age (Fig. 3B) and indicate higher FC in younger subjects. For both sex and age covariates, the significant effects on FC are mostly present in the frontal, parietal, temporal, and medial temporal lobes. Besides, significant aging effects are also present in subcortical and cerebellar regions. Twenty ROI pairs with the most significant sex or age effects on FC are listed in Table 2.

**Table 2.** ROI Pairs with the Most Significant Sex or Age Effects on FC

*For sex effects*		*For age effects*	
*ROI pair*	$- lo{g_{10}}p$	*ROI pair*	$- lo{g_{10}}p$
Temporal pole: superior temporal gyrus (left)–median cingulate and paracingulate gyri (left)	25	Cerebellum 4 and 5 (right)–hippocampus (left)	8
Temporal pole: superior temporal gyrus (left)–median cingulate and paracingulate gyri (right)	22	Inferior temporal gyrus (left)–superior frontal gyrus, dorsolateral (left)	8
Superior temporal gyrus (left)–median cingulate and paracingulate gyri (left)	21	Thalamus (right)–lenticular nucleus, putamen (left)	8
Superior temporal gyrus (right)–median cingulate and paracingulate gyri (left)	19	Lenticular nucleus, putamen (left)–caudate nucleus (left)	7
Temporal pole: superior temporal gyrus (left)–anterior cingulate and paracingulate gyri (left)	19	Inferior frontal gyrus, orbital part (left)–inferior frontal gyrus, triangular part (left)	7
Middle temporal gyrus (left)–inferior frontal gyrus, orbital part (left)	19	Inferior temporal gyrus (left)–supramarginal gyrus (left)	7
Median cingulate and paracingulate gyri (left)–precentral gyrus (left)	18	Superior temporal gyrus (left)–supramarginal gyrus (left)	7
Postcentral gyrus (left)–median cingulate and paracingulate gyri (left)	18	Inferior temporal gyrus (left)–inferior parietal, but supramarginal and angular gyri (left)	6
Superior temporal gyrus (left)–median cingulate and paracingulate gyri (right)	17	Lenticular nucleus, putamen (left)–caudate nucleus (right)	6
Median cingulate and paracingulate gyri (left)–insula (right)	17	Inferior temporal gyrus (left)–inferior temporal gyrus (right)	6

*p* represents the significance of *p*-value of the covariate (sex or age) in the regression analyses.

FC, functional connectivity; ROI, region of interest.

The percentages of intralobe and interlobe FC values having significant sex effects are presented in Supplementary Figure S1. The highest ratio (43%) is located between the frontal and temporal lobes. Frontal–occipital (31%) and temporal-subcortical (28%) also exhibit high interlobe FC ratios for significant sex effects. The percentages of FC values for sex effects in the frontal, parietal, and temporal lobes range from 21% to 43%. In contrast, the percentages in the occipital, subcortical, and cerebellar regions are all less than 4%.

We also noted that the total intracranial volume (Gray matter + White matter + CSF, calculated by FreeSurfer in the HCP MR Structural pipelines) is significantly higher in males ($p  <  1E - 59$). However, including total intracranial volume as a covariate into the regression model does not change the general pattern of sex/age effects (illustrated in Supplementary Fig. S2).

**FIG. 4.**
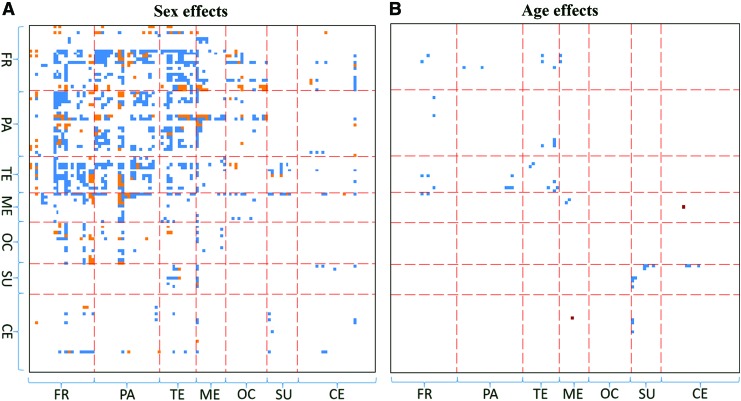
Effect of behavioral measures on sex and age effects on FC. For regression models that included seven cognitive measures as covariates, ROI pairs with significant **(A)** sex effects and **(B)** age effects on FC are presented. FR, frontal; PA, parietal; TE, temporal; ME, medial temporal; OC, occipital; SU, subcortical; CE, cerebellum. Color images available online at www.liebertpub.com/brain

Effects of sex and age on FC after adding cognitive measures to the regression models are presented in Figure 4. Compared to Figure 3A, 534 out of the 1025 ROI pairs for sex effects survive and three new pairs (AAL4—AAL87, AAL74—AAL103, and AAL86—AAL87) emerge. Regarding age effects, 21 out of the 29 ROI pairs survive and two new pairs (AAL13—AAL81 and AAL13—AAL83) emerge.

In the above regression analysis, no significant covariates for the seven cognitive measures survive Bonferroni correction at $p  <  7.49{ \rm{E}} - 6$ corresponding to α = 0.05. As an alternative, we calculated the direct correlations between the FC measures and each of the cognitive scores. The following significant associations (Bonferroni corrected, $p  <  7.49{ \rm{E}} - 6$ corresponding to α = 0.05) are found: (1) The language/vocabulary comprehension measure correlates with FC for AAL10–AAL66 and (2) The language/reading decoding measure is correlated with FC for AAL10–AAL66 and AAL16–AAL24.

### Sex and age interaction effects of functional connectivity

There were no *sex* $\times$ *age* interaction effects on FC that survive Bonferroni or false discovery rate correction. This was also confirmed by a separate ANCOVA. For regression analysis of FC versus age for males and females separately, there were no significant differences in the age regression coefficients (or the slopes) between genders. For males, not a single slope is significant after Bonferroni correction at $p  <  7.49{ \rm{E}} - 6$ corresponding to α = 0.05, while slopes for 134 (which were all negative) out of 6670 FC measures in females were significant. In this study, we explore whether the age effects on FC are qualitatively different between male and female groups.

**FIG. 5.**
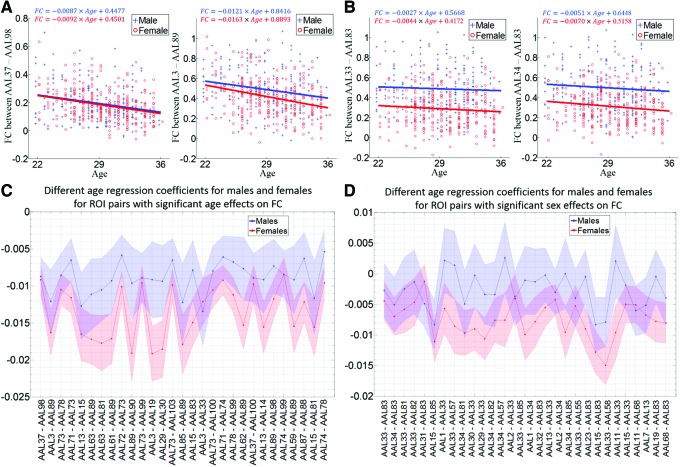
Age effects on FC for males and females separately. **(A)** Scatter plots of FC versus age for males and females, for two ROI pairs with the most significant age effects in regression analysis. **(B)** Scatter plots of FC versus age for males and females, for two ROI pairs with the most significant sex effects in regression analysis. Regression lines are drawn for males and females separately to show different age regression coefficients (different slopes of the regression lines). Different age regression coefficients for males and females for **(C)** 29 ROI pairs with significant age effects on FC and **(D)** 30 ROI pairs with significant sex effects on FC. Standard errors are shown as the shaded area. Color images available online at www.liebertpub.com/brain

In Figure 5A, B, for two ROI pairs with the most significant age or sex effects on FC in the previous regression analysis, both males and females show negative slopes and the slopes of female regression lines are larger in magnitude than males. Next, the experiment for examining different age regression coefficients between males and females was extended to all FC measures. Results for all 29 FC measures with significant age effects and 30 FC measures with the most significant sex effects in previous analysis are shown in Figure 5C, D, respectively. In Figure 5C, all the slopes are negative and the magnitude is larger for females in 27 out of 29 ROI pairs. In Figure 5D, the female slopes are still negative, while males show positive slopes in four ROI pairs. Except for three pairs in which males show a larger magnitude negative slope, male slopes are above the female slopes in all other ROI pairs: either male and female slopes are both negative or male slopes are positive, while females slopes are negative. For all ROI pairs, 3787 out of 6670 (57%) FC measures demonstrate negative age regression coefficients for both males and females and the magnitude for the female slope is larger compared to the male slope.

### Sex difference of graph properties

**FIG. 6.**
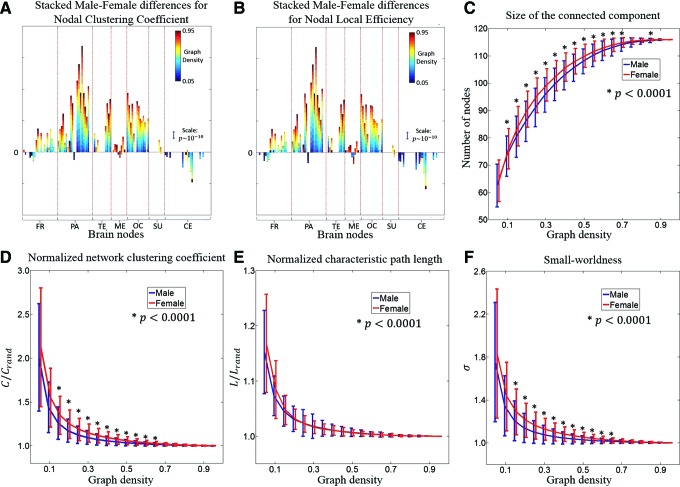
Male versus female differences for graph properties. **(A, B)** show the sex differences for the nodal clustering coefficient and the nodal local efficiency, respectively. 116 brain regions are divided into seven lobes by the red lines. The sex differences ($$sign \left( t \right) \times \left( {  -   lo{g_{10}}p} \right)$$) are color coded for different graph densities and are stacked together. Both the direction and significance of male versus female difference are displayed: above zero means male > female and below zero means female > male; the height of segment represents $$ - lo{g_{10}}p$$ where *p* is the two-sample *p*-value and the scale is given as a line segment for $$p \,\sim\, {10^{ - 10}}$$. Only significant differences ($$p  <  0.05 / 116$$) are presented and stacked. **(C)** Mean and standard deviation of the size of the connected component, which is the number of nodes in the largest connected subgraph, as a function of graph density are plotted for males and females separately. **(D–F)** Mean and standard deviation of the normalized network clustering coefficient, normalized characteristic path length, and small-worldness metric, as a function of graph density, are shown for males and females separately. Color images available online at www.liebertpub.com/brain

As shown in Figure 6A, B, sex differences for the nodal clustering coefficient and the nodal local efficiency are similar. For all lobes but the cerebellum, the stacked sex differences are in the positive side for most AAL regions. A majority of brain nodes in the cerebrum demonstrate stronger clustering coefficient and higher local efficiency in males compared to females and this result is replicated at different graph density thresholds. Sex effects in the medial temporal and the subcortical lobes are relatively weak, with significant sex differences present at only few densities. The only lobe that clearly exhibits stronger clustering coefficient and higher local efficiency in females compared to males is the cerebellum.

Across the range of graph density studied in this article (0.05–0.95), the 116 nodes in the graph may not be fully connected so there may exist isolated nodes from the major component. Therefore, we explored the size of the largest connected component (number of nodes in the component) and checked if it was different between males and females. Figure 6C illustrates that above a graph density threshold of 0.5, more than 90% of the nodes are connected. Except the densest case (0.95), females show a larger size for the connected component in all the other 18 densities, out of which 14 demonstrate a significance of $p  <  0.0001$. This result suggests that when the graphs are constructed to have the same wiring cost, the networks in female brains have more connected nodes than in males. Figure 6D–F shows male versus female group comparisons for the three global measures as a function of graph density. For both male and female, these three indices monotonically decrease and converge to 1 as the graph density increases, indicating that it becomes harder to distinguish the brain graph from its corresponding random graph at higher densities. While there is no significant male versus female difference for the normalized characteristic path length, female networks demonstrate consistently higher normalized clustering coefficient and higher small-world properties for graph densities from 0.15 to 0.65 and the two-sample *t*-test *p* values for these differences were $p < 0.0001$.

To confirm that the above two-sample *t*-test sex differences in graph measures were not influenced by age, we repeated the analysis with a regression model that contained sex and age as covariates. Effects of sex were similar to previous results and effects of age were much weaker and not consistent across graph densities (Supplementary Figs. S3 and S4).

## Discussion

### Sex effects on functional connectivity and on graph properties

One of the most significant results from our analyses is that there exist extensive sex-related differences of FC in the brain, and all FC measures that show statistically significant sex effects are greater in males than in females. Whereas most studies that note sex differences tend to have a mix of greater connectivity for either males or females (Allen et al., 2011; Filippi et al., 2013). Results of our study show that although FC differences were higher in females for certain pairs of FC measures, none of them survived multiple comparison correction. A previous study that investigated a group of healthy subjects spanning an age range similar to that of our cohort reported higher FC in the parietal and occipital regions for males compared to females (Filippi et al., 2013). This finding is replicated in our study. The increased parietal FC in men mirrors the result of fMRI studies for complex cognitive tasks (Thomsen et al., 2000), where males predominantly exhibited parietal activation. Our findings regarding increased FC in males in occipital regions are supported by Biswal et al. (2010), where FC of occipital regions is higher in males across three different methods (seed-based, fractional amplitude of low-frequency fluctuations, and ICA). Higher FC of both parietal and occipital lobes in males may possibly reflect the increased motor and visuospatial skills in men (Hamilton, 2008; Weiss et al., 2003).

**FIG. 7.**
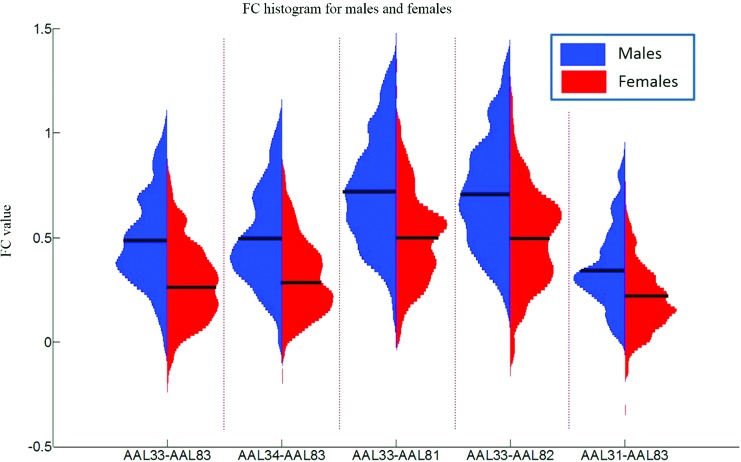
FC histograms for five ROI pairs with the most significant sex effects as listed in Table 2. Male and female FC histograms are shown vertically. The FC is derived by Fisher *z*-transforming the Pearson correlation coefficient so that the range of it is not restricted between $- 1$ and $+ 1$. The mean value of the FC distributions is indicated by a short black line. Color images available online at www.liebertpub.com/brain

Despite these robust sex differences, it should be noted that there is a large degree of overlap in FC of males and females. To illustrate this overlap, in Figure 7, we present FC histograms for five ROI pairs with the most significant sex effects. Although the average FC values for males and females are significantly different, there is a large overlap of FC values between the two groups. This male/female overlap of FC is also present across all ages, as indicated in Figure 5A, B.

While some of our results strongly replicate previous findings, several findings do not. For frontal and temporal lobes, Filippi et al. (2013) reported stronger female FC, but we show the opposite trend (male >female). fMRI studies of language processing have indicated that, in comparison to males, females tend to have a higher bilateral activation in the frontal and temporal, when females had higher language performance scores (Baxter et al., 2003; Kansaku et al., 2000). The distinction regarding the directionality of sex differences may be attributed to the fact that in our cohort, two scores related to language processing are significantly higher in males (Table 1), which is unlike previous studies. Furthermore, previous studies did not use whole-brain AAL parcellations to calculate FC and this may be another reason for disparate findings. However, the association between language performance and frontal and temporal FC requires further investigation.

In a separate analysis, we evaluated the effect of cognitive measures on sex differences of FC. After the seven cognitive measures were incorporated into the regression models, the main pattern of the sex effects for the frontal, parietal, and temporal lobes in Figure 3A remained significant (Fig. 4A). This result was true for the age effects as well as we observed little change after adding the cognitive scores to the regression model. Therefore, we conclude that the sex and age effects on FC are robust to the cognitive measures.

With regard to the sex effect of graph measures, we first examined the size of the connected graph (Fig. 6C). Bassett et al. (2012) reported that this metric, defined as the number of nodes in the largest connected component, was significantly correlated with more complex graph measures (e.g., global efficiency or betweenness centrality) in a wide range of graph densities. Therefore, the size of the brain network may be an important indicator of the underlying topology. Our findings that female functional networks have significantly more connected nodes than males suggest an increased network homogeneity in female brains.

For the regional graph properties, we observed that nodes in the cerebellum have higher clustering coefficient and local efficiency for females, while nodes at other lobes show sex difference in the opposite direction. The most significant difference is in the parietal and occipital lobes, which may add credence to sex differences in FC discussed earlier. Results regarding the regional graph properties are in agreement with one diffusion imaging study (Ingalhalikar et al., 2014), in which the cerebellum was the only region that displayed higher participation coefficients in males, while in all other lobes, the cross-module participation dominated in females. Since higher participation coefficients indicate that connections are more uniformly distributed among the lobes, the diffusion study illustrated that in males, for all lobes but the cerebellum, FC is more focused within each module. This adds evidence to our findings of higher clustering coefficient and local efficiency in males, while the female connections are more spread between lobes and the network is less modular. This result and the larger size of the female brain networks jointly support the notion that female brain networks, compared to male networks, are more spatially distributed but at lower correlation strengths.

For the global properties of the graph, the normalized network clustering coefficient and the normalized characteristic path length were derived to calculate the small-world metric. While males and females do not differ in the characteristic path length, the normalized network clustering coefficient for females is significantly higher in a wide range of densities. This is consistent with the results in Yan et al. (2011) and makes the small-world metric to be higher for female networks. While both male and female brains clearly demonstrate small-world characteristics, there exist differences in the trade-off between local segregation and global integration of the network topology. We observed that male brains prevail in functional segregation, while female brains facilitate functional integration. Combining the effect of sex on FC and on graph metrics, we hypothesize that males are more likely better at performing a single task, whereas females are more equipped for performing multiple tasks, as has also been supported in a study using DTI (Ingalhalikar et al., 2014).

### Age effects on functional connectivity

Age effects on FC have been explored in various studies. Among the findings, the extensive involvement of the medial temporal regions in age effects has been previously reported (Chou et al., 2013; Jacques, 2009; Li et al., 2014). However, only a few medial temporal regions (temporal pole and hippocampus) are present for age effects in our analysis. Given that the medial temporal lobe plays an important role in human memory (Buckner, 2004), we hypothesize that the medial temporal regions reported in studies involving large age ranges are due to significant memory deficits observed in later life. In our study, correlations between age and episodic/working memories are not significant and this adds evidence to the above hypothesis.

In our analyses, age effects on FC within cortical regions are mostly present in frontal, parietal, and temporal lobes. The effect of age on these three lobes has been reported in previous studies. Steffener et al. (2012) reported decreased FC in older adults between the supplementary motor area and the middle cingulate and between the precuneus and the middle/superior frontal cortex. Campbell et al. (2012) revealed reduced FC within the frontoparietal network in older adults, suggesting decreased activity and coherence within a putative control network. Also, the DMN, for which most components are in frontal or parietal lobes, has been consistently demonstrated to be susceptible to aging (Andrews-Hanna et al., 2007; Damoiseaux et al., 2008; Ferreira and Busatto, 2013; Grady, 2012). Our analyses detect age-related FC reductions in the medial prefrontal cortex, hippocampus, and inferior parietal gyrus, which are components of the DMN. This provides evidence that DMN regions are subject to aging, even in early adulthood. Finally, Campbell et al. report lower FC for older subjects in insula, superior temporal, middle temporal, and inferior temporal regions.

Besides the cortical lobes, our findings of age effects for the subcortical and cerebellar regions are also in line with previous studies. The caudate, putamen, and pallidum (Bollinger et al., 2011), which together constitute the basal ganglia, have been reported to demonstrate age effect on FC. Given that age-related differences in functional activation of the basal ganglia have been consistently reported during cognitive and motor tasks (Rubia et al., 2007; Wu and Hallett, 2005), the presence of reduced FC between putamen and caudate in our results indicates that the basal ganglia can be a robust marker for age effects. The role of the cerebellum in normal aging has been reported, where cerebellum–striatum and cerebellum–medial temporal lobe FC disruptions were noted (Bernard et al., 2013). This matches well with our results where FC between cerebellar regions and putamen/caudate (part of the dorsal striatum) and the hippocampus (part of the medial temporal lobe) is lower for older subjects. Bernard et al. (2013) suggested that the decreased striatal–cerebellar and hippocampus–cerebellar FC may be attributed to reduced dopamine levels and deficits in memory/associative learning in normal aging, respectively.

### Application of the AAL atlas as a limitation

Although FC derivations based on anatomical ROIs have been explored extensively using different parcellation schemes (Craddock et al., 2012; de Reus and van den Heuvel, 2013; Marrelec and Fransson, 2011; Poldrack, 2007; Shirer et al., 2012), researchers have become more aware that functional inhomogeneity for anatomical parcellation may induce biases for network construction and graph analyses.

To determine the extent to which the average AAL time-courses were representative of the respective AAL regions, we calculated the number of voxels that were significantly correlated (*p* < 0.05) with the average time series within each AAL region. Out of 116 AAL regions, we found that 63 regions (54%) had more than 50% of voxels with significant correlations to the average time series. This indicates that using the AAL atlas for functional parcellation is reasonable.

The AAL atlas parcellation helped to locate significant sex and age effects on FC. However, it performed poorly in identifying associations with behavioral measures (see Sex and Age Effects of Functional Connectivity). In future studies, we intend to explore FC and FC associations to behavioral measures using other parcellations.

## Conclusion

In this study, we demonstrated significant sex and age effects in early adulthood for healthy subjects using full brain resting-state FC. Our findings indicate widespread sex effects in which males exhibit higher FC than females for all significant measures. For the much less widespread brain regions associated with age effects, the involvement of some systems (e.g., DMN, basal ganglia) match well with findings in previous studies, which spanned larger age ranges, therefore suggesting robust markers for aging. Graph measures using a proportional threshold scheme demonstrate that both male and female brains exhibit small-world characteristics but with subtle significant differences in the organization of the networks. While male brains generally have higher clustering coefficient and higher local efficiency at the nodes of the graph, female brains are more connected at the whole-brain level. These findings illustrate the necessity of including sex and age as covariates in future fMRI studies and provide evidence that brain networks show male/female differences. The sex differences of FC indicate that male brain networks show signs of segregation and that female brain networks show signs of integration.

## Supplementary Material

Supplemental data

Supplemental data

Supplemental data

Supplemental data

Supplemental data
