# Supplementary material for: Sex and Age Effects of Functional Connectivity in Early Adulthood
Source: Brain Connect. 2016 Nov 1;6(9):700–13. doi: 10.1089/brain.2016.0429 (PMC5105352; doi:10.1089/brain.2016.0429)
Supplement: Supplemental data [file Supp_Table1.pdf]

Supplementary Table S1. AAL Atlas Regions

AAL1-2	Precentral gyrus (left and right)	AAL63-64	Supramarginal gyrus
AAL3-4	Superior frontal gyrus, dorsolateral	AAL65-66	Angular gyrus
AAL5-6	Superior frontal gyrus, orbital part	AAL67-68	Precuneus
AAL7-8	Middle frontal gyrus	AAL69-70	Paracentral lobule
AAL9-10	Middle frontal gyrus orbital part	AAL71-72	Caudate nucleus
AAL11-12	Inferior frontal gyrus, opercular part	AAL73-74	Lenticular nucleus, putamen
AAL13-14	Inferior frontal gyrus, triangular part	AAL75-76	Lenticular nucleus, pallidum
AAL15-16	Inferior frontal gyrus, orbital part	AAL77-78	Thalamus
AAL17-18	Rolandic operculum	AAL79-80	Heschl gyrus
AAL19-20	Supplementary motor area	AAL81-82	Superior temporal gyrus
AAL21-22	Olfactory cortex	AAL83-84	Temporal pole: superior temporal gyrus
AAL23-24	Superior frontal gyrus, medial	AAL85-86	Middle temporal gyrus
AAL25-26	Superior frontal gyrus, medial orbital	AAL87-88	Temporal pole: middle temporal gyrus
AAL27-28	Gyrus rectus	AAL89-90	Inferior temporal gyrus
AAL29-30	Insula	AAL91-92	Cerebellum crus 1
AAL31-32	Anterior cingulate and paracingulate gyri	AAL93-94	Cerebellum crus 2
AAL33-34	Median cingulate and paracingulate gyri	AAL95-96	Hemispheric lobule 3
AAL35-36	Posterior cingulate gyrus	AAL97-98	Hemispheric lobule 4/5
AAL37-38	Hippocampus	AAL99-100	Hemispheric lobule 6
AAL39-40	Parahippocampal gyrus	AAL101-102	Hemispheric lobule 7B
AAL41-42	Amygdala	AAL103-104	Hemispheric lobule 8
AAL43-44	Calcarine fissure and surrounding cortex	AAL105-106	Hemispheric lobule 9
AAL45-46	Cuneus	AAL107-108	Hemispheric lobule 10
AAL47-48	Lingual gyrus	AAL109	Vermic lobule 1/2
AAL49-50	Superior occipital gyrus	AAL110	Vermic lobule 3
AAL51-52	Middle occipital gyrus	AAL111	Vermic lobule 4/5
AAL53-54	Inferior occipital gyrus	AAL112	Vermic lobule 6
AAL55-56	Fusiform gyrus	AAL113	Vermic lobule 7
AAL57-58	Postcentral gyrus	AAL114	Vermic lobule 8
AAL59-60	Superior parietal gyrus	AAL115	Vermic lobule 9
AAL61-62	Inferior parietal, but supramarg and angular gyri	AAL116	Vermic lobule 10

From AAL1 to AAL108, odd/even number corresponds to left/right part.
