# Supplementary material for: Sex and Age Effects of Functional Connectivity in Early Adulthood
Source: Brain Connect. 2016 Nov 1;6(9):700–13. doi: 10.1089/brain.2016.0429 (PMC5105352; doi:10.1089/brain.2016.0429)
Supplement: Supplemental data [file Supp_Fig1.pdf]

Supplementary Data

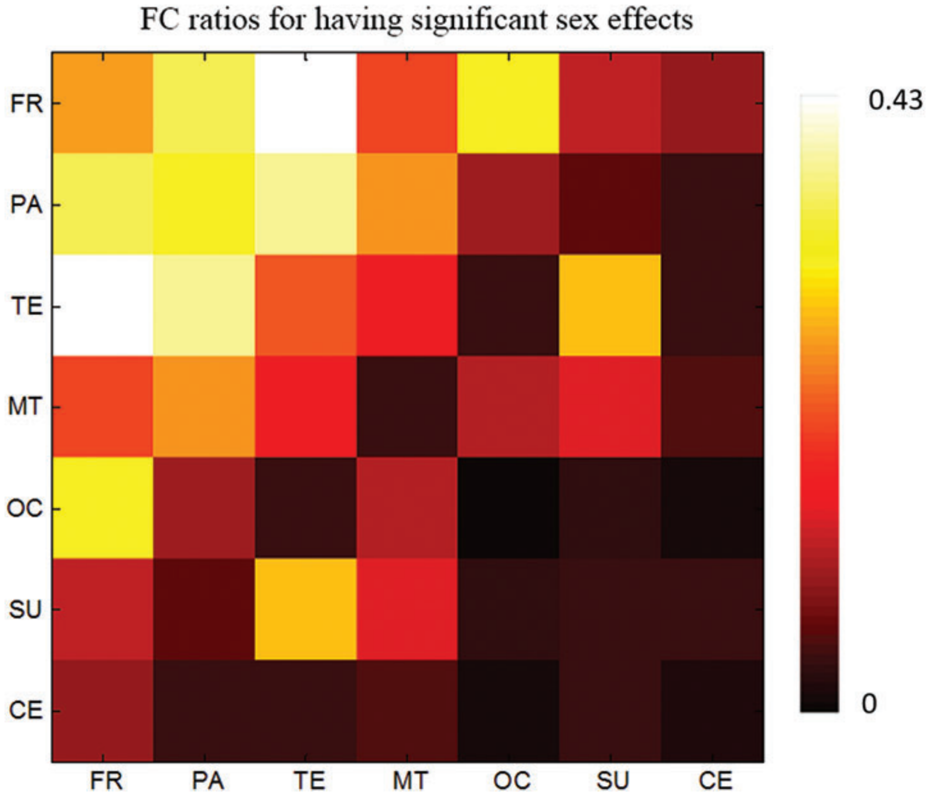

SUPPLEMENTARY FIG. S1. FC ratios for having significant sex effects. Each entry is the ratio between the number of significant intra/interlobe ROI pairs divided by total number of intra/interlobe ROI pairs. FR, frontal; PA, parietal; TE, temporal; ME, medial temporal; OC, occipital; SU, subcortical; CE, cerebellum; FC, functional connectivity; ROI, region of interest.
