# Supplementary material for: Sex and Age Effects of Functional Connectivity in Early Adulthood
Source: Brain Connect. 2016 Nov 1;6(9):700–13. doi: 10.1089/brain.2016.0429 (PMC5105352; doi:10.1089/brain.2016.0429)
Supplement: Supplemental data [file Supp_Fig2.pdf]

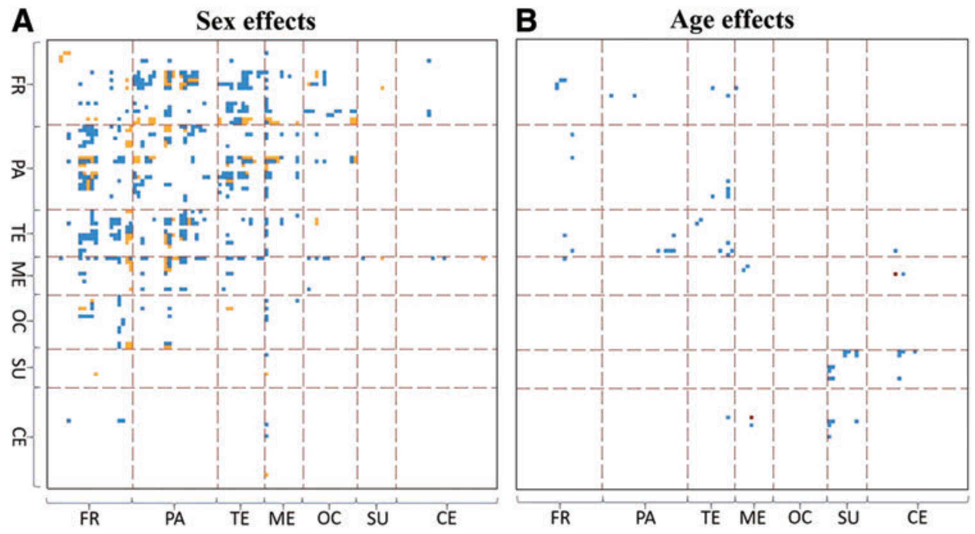

SUPPLEMENTARY FIG. S2. After adding the total intracranial volume (Gray Matter+White Matter+CSF volume) as an additional covariate into the regression model, ROI pairs with significant **(A)** sex effects and **(B)** age effects ($p < 7.49E - 6$ corresponding to Bonferroni threshold at $\alpha = 0.05$) on FC are presented in the FC matrix. In the matrix plots, ROI pairs with significant sex and age effects are color coded (as in Fig. 3) to indicate the model from which the significance of the covariates are derived. FR, frontal; PA, parietal; TE, temporal; ME, medial temporal; OC, occipital; SU, subcortical; CE, cerebellum.
