# Supplementary material for: Sex and Age Effects of Functional Connectivity in Early Adulthood
Source: Brain Connect. 2016 Nov 1;6(9):700–13. doi: 10.1089/brain.2016.0429 (PMC5105352; doi:10.1089/brain.2016.0429)
Supplement: Supplemental data [file Supp_Fig3.pdf]

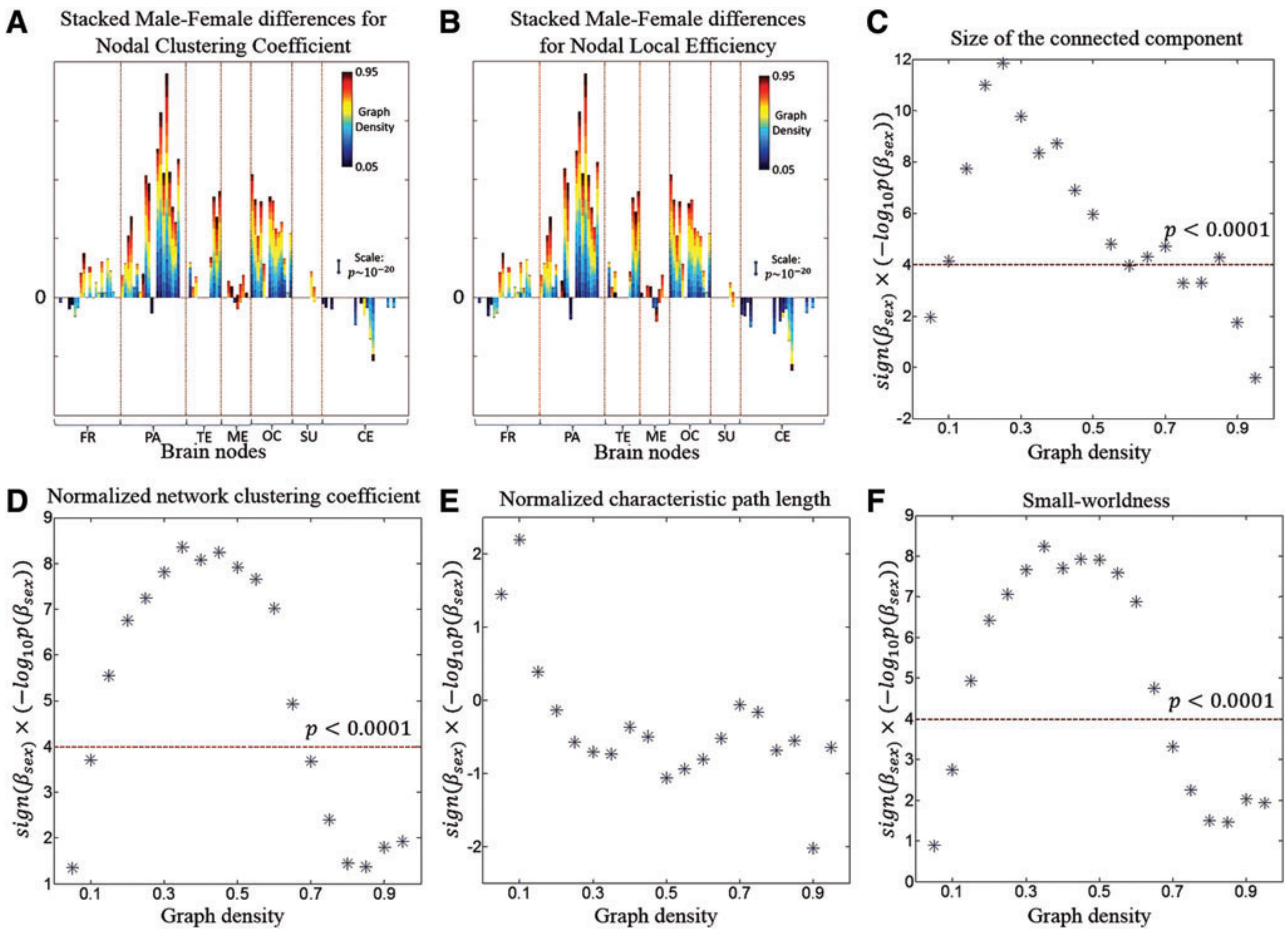

SUPPLEMENTARY FIG. S3. Sex effects of graph properties by regression model. **(A, B)** show the sex differences for the nodal clustering coefficient and the nodal local efficiency, respectively. 116 brain regions are divided into seven lobes by the red lines. The sex differences $sign(\beta_{sex)} \times (-log_{10}p(\beta_{sex}))$ are color coded for different graph densities and are stacked together. Both the direction and significance of male versus female difference are displayed (Note: the signs of the regression efficient are reversed for comparison with Fig. 6): above zero means male>female and below zero means female>male; the height of segment represents $-log_{10}p$ where p is the significance of regression coefficient for sex covariate and the scale is given as a line segment for $p \sim 10^{-20}$. Only significant differences ($p < 0.05/116$) are presented and stacked. **(C–F)**: Sex effect across graph densities for **(C)** size of graph; **(D)** normalized network clustering coefficient; **(E)** normalized characteristic path length; and **(F)** small-worldness metric. For **(C–F)**, positive indicates female>male and negative indicates male>female. The red dashed line demonstrates the threshold of $p < 0.0001$. Here, significant sex differences are present only for some positive points in **(C, D, F)** where the graph measure is larger in females.
