# Supplementary material for: Sex and Age Effects of Functional Connectivity in Early Adulthood
Source: Brain Connect. 2016 Nov 1;6(9):700–13. doi: 10.1089/brain.2016.0429 (PMC5105352; doi:10.1089/brain.2016.0429)
Supplement: Supplemental data [file Supp_Fig4.pdf]

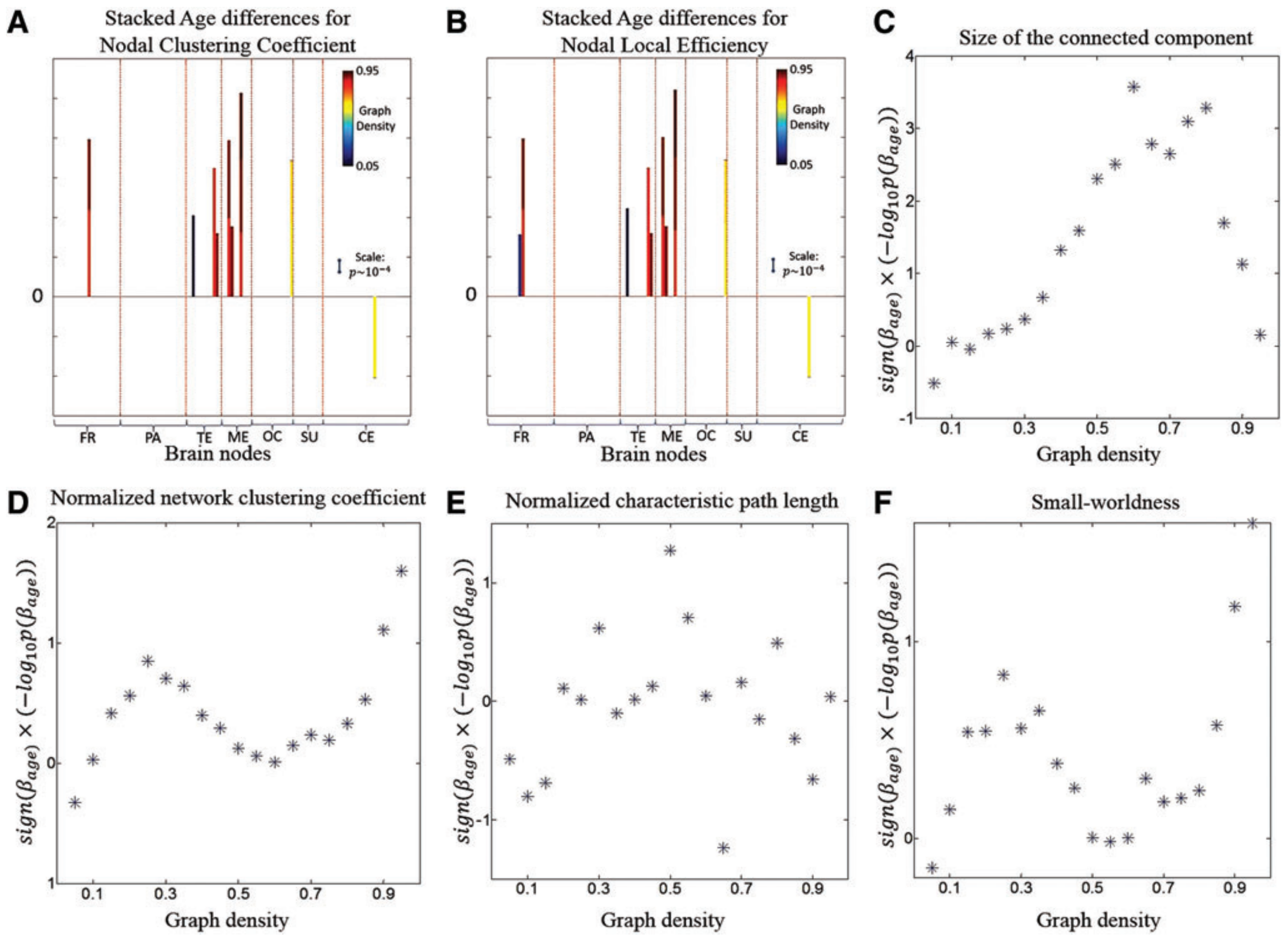

SUPPLEMENTARY FIG. S4. Age effects of graph properties by regression model. **(A, B)** show the age differences for the nodal clustering coefficient and the nodal local efficiency, respectively. 116 brain regions are divided into seven lobes by the red lines. The age differences $sign(\beta_{age}) \times \left(-log_{10}p\left(\beta_{age}\right)\right)$ are color coded for different graph densities and are stacked together. Both the direction and significance of age difference are displayed (Note: the signs of the regression efficient are reversed): above zero means decreasing across age and below zero means increasing across age; the height of segment represents $-log_{10}p$ where p is the significance of regression coefficient for age covariate and the scale is given as a line segment for $p{\sim}10^{-4}$. Only significant differences ($p < 0.05/116$) are presented and stacked. **(C–F)** Age effect across graph densities for **(C)** size of graph; **(D)** normalized network clustering coefficient; **(E)** normalized characteristic path length; and **(F)** small-worldness metric. For **(C–F)**, positive indicates increasing across age and negative indicates decreasing across age. Here, no significant age differences ($p < 0.0001$) are present for the global graph measures (no point is larger than 4 or smaller than –4).
